# Human Metapneumovirus Versus Respiratory Syncytial Virus in the Pediatric Emergency Department: Inflammatory Indices, Cycle‐Threshold Profiles and Outcomes

**DOI:** 10.1002/jmv.71099

**Published:** 2026-08-10

**Authors:** Yılmaz Seçilmiş, Gülşah Kartal, Muhammet Sami Kayan, Mesut Kara, Ömür Mustafa Parkan, Selma Gökahmetoğlu

**Affiliations:** ^1^ Department of Pediatrics, Division of Pediatric Emergency Faculty of Medicine, Erciyes University Kayseri Türkiye; ^2^ Department of Pediatrics Faculty of Medicine, Erciyes University Kayseri Türkiye; ^3^ Department of Medical Microbiology Faculty of Medicine, Erciyes University Kayseri Türkiye

**Keywords:** antimicrobial stewardship, cycle threshold, human metapneumovirus, multiplex RT‐PCR, neutrophil‐to‐lymphocyte ratio, pediatric emergency medicine, respiratory syncytial virus, systemic immune‐inflammation index, viral co‐detection

## Abstract

Inflammatory‐biomarker, cycle‐threshold (Ct) and co‐detection data on human metapneumovirus (hMPV) in the pediatric emergency department remain scarce. We retrospectively studied 203 children with multiplex RT‐PCR–confirmed respiratory virus positivity at a tertiary pediatric emergency department (January–May 2025). Inflammatory indices, C‐reactive protein (CRP) and per‐target Ct were compared between hMPV and RSV; predictors of antibiotic prescribing were assessed by ROC and multivariable regression. hMPV (17.7%) and RSV (28.6%) were dominant detections; co‐detection 24.1%. Compared with RSV, hMPV affected older children with higher comorbidity, more pneumonia and neutrophil‐driven inflammation (neutrophil‐to‐lymphocyte ratio [NLR], systemic immune‐inflammation index [SII], CRP higher; *p* ≤ 0.006). Dominant Ct differed across pathogens (Kruskal–Wallis *p* < 0.001); rhinovirus carried the lowest viral load. Paired non‐dominant Ct exceeded dominant Ct (median ΔCt +3.6, *p* = 0.013). CRP independently predicted antibiotic prescribing (AUC 0.78, cut‐off 3.9 mg/L). hMPV showed a neutrophil‐dominant inflammatory phenotype in older, more comorbid children than RSV. Per‐target Ct supports the lowest‐Ct‐dominant convention. CRP—not white‐cell indices—drove antibiotic prescribing; a 3.9 mg/L cut‐off supports stewardship in PCR‐confirmed pediatric viral respiratory infection.

## 1 Introduction

Human metapneumovirus (hMPV), a negative‐sense single‐stranded RNA virus of the family Pneumoviridae first described by van den Hoogen and colleagues in 2001, is now recognized as a major cause of acute lower respiratory tract infection (LRTI) in children and is second only to respiratory syncytial virus (RSV) as a reason for viral LRTI hospitalization in infants and young children worldwide [1, 2, 3, 4, 5]. Global disease‐burden estimates suggest that hMPV is responsible for approximately one in ten pediatric LRTI hospitalizations and nearly 16,000 under‐5 deaths annually, with the heaviest burden in low‐ and middle‐income countries [6, 7, 8]. Its clinical presentation overlaps with that of RSV and influenza, and the increasing adoption of multiplex reverse‐transcription polymerase chain reaction (RT‐PCR) respiratory panels in emergency departments (EDs) has transformed detection of hMPV and characterization of the viral landscape in which it circulates, including high rates of viral co‐detection [9, 10, 11, 12].

Three clinically consequential knowledge gaps persist. First, although hMPV has traditionally been positioned as a “junior RSV,” emerging data indicate that the two viruses elicit divergent innate‐immune responses, with hMPV typically affecting older children and showing a distinct neutrophilic signature [13, 14, 15, 16]. Systemic inflammatory indices derived from the routine complete blood count—neutrophil‐to‐lymphocyte ratio (NLR), platelet‐to‐lymphocyte ratio (PLR) and the composite Systemic Immune‐Inflammation Index (SII = platelets × neutrophils/lymphocytes)—offer a low‐cost, reproducible window on innate immune tone [17, 18, 19, 20, 21]. Their systematic comparison across pediatric respiratory viral pathogens in the ED is, however, limited.

Second, in the era of multiplex testing, viral co‐detection is identified in roughly one in five children with acute respiratory infection [9, 22, 23, 24], but its clinical meaning remains contested. Some reports suggest co‐detection aggravates illness, others find it clinically neutral, and a third group conclude that it chiefly reflects prolonged post‐infectious shedding of one of the detected agents [8, 22, 23, 24]. Quantitative cycle‐threshold (Ct) data—available from most multiplex platforms as a semi‐quantitative surrogate of viral load [25, 26, 27] —offer a mechanistic lever to adjudicate which virus in a co‐detection is biologically dominant, yet pediatric ED studies rarely report per‐target Ct and paired dominant‐vs‐non‐dominant comparisons.

Third, decisions about hospitalization and antibiotic prescribing in pediatric viral respiratory infection rest on a mosaic of clinical syndrome, vital signs and low‐cost laboratory biomarkers. CRP‐guided point‐of‐care algorithms have reduced antibiotic exposure in adult and pediatric primary care settings without increasing complications [28, 29, 30, 31], but the relative contributions of CRP, inflammatory indices, Ct and clinical diagnosis within a PCR‐confirmed pediatric viral population have not been systematically quantified.

We therefore conducted a contemporary single‐center retrospective cohort study of consecutive PCR‐confirmed viral respiratory infections in a tertiary pediatric ED across a complete January–May 2025 epidemic season. Our pre‐specified objectives were to (i) describe the epidemiology of hMPV and concurrent respiratory viruses; (ii) characterize NLR, PLR, SII, CRP and dominant‐target Ct across the five most prevalent pathogens and specifically compare hMPV with RSV; (iii) map viral co‐detection patterns and quantify the dominant‐vs‐non‐dominant Ct differential; (iv) identify pre‐specified laboratory and clinical predictors of hospitalization and antibiotic prescribing in hMPV‐positive children; and (v) derive an actionable CRP cut‐point for antimicrobial stewardship.

## 2 Materials and Methods

### 2.1 Study Design and Setting

We performed a single‐center retrospective cohort study at the pediatric ED of Erciyes University Faculty of Medicine, a tertiary academic hospital with ~60,000 pediatric ED visits/year. The study period (1 January–31 May 2025) covers the complete late‐winter/spring respiratory viral epidemic season, including the hMPV peak. Reporting follows the STROBE statement [32] (Supporting Information Checklist S1). The Erciyes University Health Sciences Research Ethics Committee approved the study (approval no. 2026/184, 08.04.2026); a waiver of written consent was granted given the retrospective, de‐identified nature of the data. The study adhered to the Declaration of Helsinki.

### 2.2 Participants and Selection Criteria

Eligible participants were consecutive children aged 0–18 years presenting to the pediatric ED with acute respiratory tract infection and in whom a nasopharyngeal swab was tested by multiplex RT‐PCR as part of routine care. Inclusion criteria: symptom duration ≤ 14 days, ≥ 1 respiratory virus detected, and complete core clinical and laboratory data at ED presentation. Exclusion criteria: antiviral therapy within the preceding 48 h, confirmed non‐infectious etiology, and transfer from another facility. Patients missing complete blood count or CRP were excluded from the main analysis (Supporting Information Figure S1).

### 2.3 Clinical Definitions

Bronchiolitis was diagnosed per American Academy of Pediatrics (AAP) criteria [33]. Pneumonia was defined as physician‐diagnosed LRTI supported by clinical findings (fever, cough, tachypnoea, abnormal auscultation) and consistent imaging (alveolar or interstitial infiltrate on chest radiograph), applying British Thoracic Society pediatric pneumonia criteria [34]. Upper respiratory tract infection (URTI) included acute pharyngotonsillitis, acute otitis media, laryngotracheitis and nasopharyngitis. Disease severity at ED disposition was classified prospectively into mild, moderate and severe categories by the attending pediatric emergency physician using a pre‐specified composite score encompassing oxygen requirement, work of breathing (Silverman–Andersen retraction score) and oral feeding tolerance.

### 2.4 Multiplex RT‐PCR, Cycle Threshold and Co‐Detection Classification

Nasopharyngeal swabs were collected in universal transport medium and tested within 4 h of ED presentation using the QIAstat‐Dx Respiratory Panel (QIAGEN, Hilden, Germany), a validated commercial multiplex RT‐PCR assay (targets: influenza A [including subtyping], influenza B, RSV subtypes A/B, parainfluenza 1–4, hMPV, rhinovirus/enterovirus, adenovirus, bocavirus, SARS‐CoV‐2, and the seasonal human coronaviruses HKU1, OC43, NL63 and 229E). Per manufacturer validation, a sample was considered positive at Ct < 40 with concordant internal positive‐control amplification. Co‐detection was defined *a priori* as simultaneous positive amplification of ≥ 2 distinct viral targets on the same sample, with up to four targets recorded per sample. Ct values for each positive target were retrieved from the microbiology laboratory information system, sourced directly from the QIAstat‐Dx instrument, as they were not available in clinical charts. When multiple viruses were detected in a single patient, we designated as the dominant target the one with the lowest Ct (i.e., highest viral load), and as non‐dominant targets the remaining viruses; this lowest‐Ct‐dominant convention was pre‐specified and empirically tested in §3.5 using a paired Wilcoxon comparison of dominant and non‐dominant Ct values.

### 2.5 Laboratory Analyzes and Inflammatory Indices

A complete blood count (Sysmex XN‐1000 platform) and high‐sensitivity turbidimetric CRP (Roche Cobas c‐501) were obtained at ED presentation. NLR was calculated as absolute neutrophil count/absolute lymphocyte count; PLR as platelet count/absolute lymphocyte count; and SII as (platelets × neutrophils)/lymphocytes, all with platelets, neutrophils and lymphocytes expressed in 10^9^/L units. Mean platelet volume (MPV) and hemoglobin were additionally recorded.

### 2.6 Sample Size Considerations

This was a cohort‐defined study (all eligible ED visits in the defined epidemic season). The enrolled sample (*n* = 203) provided ≥ 80% power (two‐sided α = 0.05) to detect a between‐group NLR shift of 0.5 units (hMPV vs. RSV) and a CRP‐based AUC ≥ 0.75 versus the null of 0.50, as confirmed by post‐hoc simulation.

### 2.7 Statistical Analysis

Continuous variables are reported as median (IQR) and compared by Mann–Whitney U (two groups) or Kruskal–Wallis (≥ 3 groups). Categorical variables are reported as frequencies with exact 95% CIs (Clopper–Pearson) and compared by chi‐square or Fisher's exact test. The hMPV‐vs‐RSV contrast was the pre‐specified primary comparison; remaining between‐pathogen tests were Benjamini–Hochberg FDR‐corrected (q < 0.05) and labeled exploratory. Paired dominant‐vs‐non‐dominant Ct values were compared by Wilcoxon signed‐rank test. In hMPV‐positive children, associations of CRP, NLR, SII, WBC, age and dominant Ct with hospitalization and antibiotic prescribing were assessed by univariable logistic regression and ROC analysis with Youden‐derived cut‐points and 2000‐resample bootstrap 95% CIs. A pre‐specified multivariable model included age, pneumonia diagnosis and CRP (events‐per‐variable ≈4.7); a sensitivity model added dominant Ct. Model adequacy was assessed by likelihood‐ratio test and McFadden pseudo‐R^2^. Two‐sided *p* < 0.05 was considered significant. Analyzes used Python 3.11 (scipy, statsmodels, scikit‐learn, pyreadstat) and R 4.3. Missing data were handled by complete‐case analysis.

## 3 Results

### 3.1 Cohort Characteristics and Pathogen Distribution

During the 5‐month study window, 203 children met the inclusion criteria and entered the analysis (Supporting Information Figure S1). Median age was 7 months (IQR 2–18 months); 116/203 (57.1%) were male; 71/203 (35.0%) had at least one recognized comorbidity. RSV was the most prevalent dominant pathogen (58/203, 28.6%, 95% CI 22.5–35.3%), followed by rhinovirus (43/203, 21.2%, 95% CI 15.8–27.5%), hMPV (36/203, 17.7%, 95% CI 12.7–23.7%), influenza A (26, 12.8%), adenovirus (11, 5.4%), influenza B (10, 4.9%), parainfluenza (7, 3.4%), seasonal human coronaviruses combined (10, 4.9%), bocavirus (1, 0.5%) and SARS‐CoV‐2 (1, 0.5%) (Supporting Information Figure S2).

### 3.2 Viral Co‐Detection Patterns

**Figure 1:**
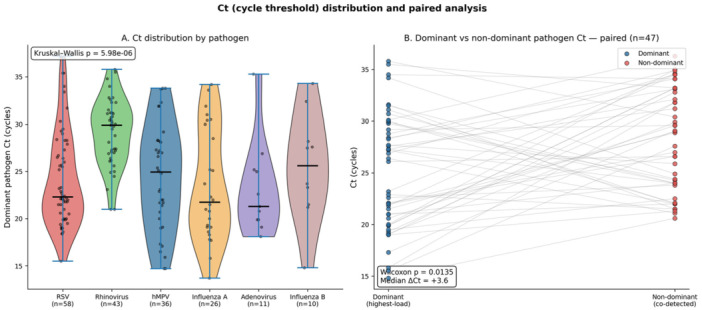
Cycle‐threshold (Ct) distribution by pathogen and paired dominant‐vs‐non‐dominant Ct. (A) Violin‐plot distribution of dominant‐target Ct across the six most prevalent pathogens; median shown as internal black bar; jitter points show individual values. Kruskal–Wallis *p* = 2 × 10^−6^. (B) Paired dominant (lower Ct) versus non‐dominant (higher Ct) values from the same patient's multiplex panel (*n* = 47 pairs); lines connect paired values. Median ΔCt +3.6 cycles; Wilcoxon signed‐rank *p* = 0.013.

A viral co‐detection was present in 49/203 children (24.1%, 95% CI 18.3–30.8%): dual in 42 (85.7% of co‐detections), triple in 2 (4.1%) and quadruple in 5 (10.2%). The commonest co‐detection pairs were RSV + rhinovirus (*n* = 12), seasonal coronavirus HKU1 + RSV (*n* = 6), RSV + influenza A (*n* = 4), hMPV + RSV (*n* = 4), rhinovirus + influenza A (*n* = 4) and hMPV + rhinovirus (*n* = 3) (Supporting Information Figure S3). Co‐detection was more frequent in rhinovirus‐positive children (15/43, 34.9%) than in RSV (14/58, 24.1%) or hMPV (8/36, 22.2%) primary infections. Paired dominant‐vs‐non‐dominant Ct comparisons across all co‐detected pairs (*n* = 47 with both targets' Ct available) showed that non‐dominant pathogens carried a significantly higher Ct (median 28.9 vs. 26.1 for the dominant target; median ΔCt +3.6 cycles, Wilcoxon signed‐rank *p* = 0.013; Figure 1B), empirically supporting the lowest‐Ct convention for designating the dominant pathogen.

### 3.3 Demographic and Clinical Characteristics: hMPV Versus RSV

Of 36 hMPV‐positive children, 21 (58.3%) were male and median age was 18 months (IQR 7–72), significantly older than RSV‐positive children (median 2 months, IQR 1–6; *p* < 0.001; Table 1). The age distribution was bimodal in hMPV, with 36.1% aged < 12 months and 27.8% aged ≥ 60 months—a distribution essentially absent in RSV (< 12 months 89.7%; ≥ 60 months 0%, both *p* < 0.001). For comparison, rhinovirus‐positive children (*n* = 43) showed an intermediate age profile (median 10 months, IQR 3–14.5; < 12 months 58.1%, 12–59 months 32.6%, ≥ 60 months 9.3%), distinct from both hMPV (Mann–Whitney *p* = 0.007) and RSV (*p* < 0.001). Any comorbidity was significantly more frequent in hMPV (50.0% vs. 22.4%; *p* = 0.007). Pneumonia was the commonest clinical diagnosis in hMPV (13/36, 36.1%), whereas bronchiolitis dominated in RSV (46/58, 79.3%); URTI presentations were more frequent in hMPV. Symptom duration at presentation was shorter in hMPV than in RSV (median 2 vs. 3 days, *p* = 0.003).

**Table 1:** Demographic and clinical characteristics of hMPV‐positive, RSV‐positive and rhinovirus‐positive children (*N* = 137).

Characteristic	hMPV (*n* = 36)	RSV (*n* = 58)	Rhinovirus (*n* = 43)	*p* *
Age, months, median (IQR)	18 (7–72)	2 (1–6)	10 (3–14.5)	< 0.001
< 12 months, *n* (%)	13 (36.1)	52 (89.7)	25 (58.1)	< 0.001
12–59 months, *n* (%)	13 (36.1)	6 (10.3)	14 (32.6)	0.001
≥ 60 months, *n* (%)	10 (27.8)	0 (0.0)	4 (9.3)	< 0.001
Male sex, *n* (%)	21 (58.3)	29 (50.0)	27 (62.8)	0.42
Any comorbidity, *n* (%)	18 (50.0)	13 (22.4)	16 (37.2)	0.021
Symptom duration, days, median (IQR)	2 (1–3)	3 (2–4)	2 (1–3)	0.002
Any viral co‐detection, *n* (%)	8 (22.2)	14 (24.1)	15 (34.9)	0.37
Diagnosis, *n* (%)				
Bronchiolitis	10 (27.8)	46 (79.3)	24 (55.8)	< 0.001
Pneumonia	13 (36.1)	7 (12.1)	6 (14.0)	0.009
URTI/other	13 (36.1)	5 (8.6)	13 (30.2)	0.003

Abbreviations: IQR, interquartile range; URTI, upper respiratory tract infection.

* Three‐group comparison (hMPV vs. RSV vs. rhinovirus) by Kruskal–Wallis (continuous) or χ^2^/Fisher's exact test (categorical). For age, pairwise post‐hoc Mann–Whitney U (uncorrected): hMPV versus RSV *p* < 0.001; hMPV versus rhinovirus *p* = 0.007; RSV versus rhinovirus *p* < 0.001. The diagnosis sub‐rows are tested individually (binary indicator vs. all other diagnoses).

### 3.4 Inflammatory Biomarker Profiles Across Pathogen Groups

**Figure 2:**
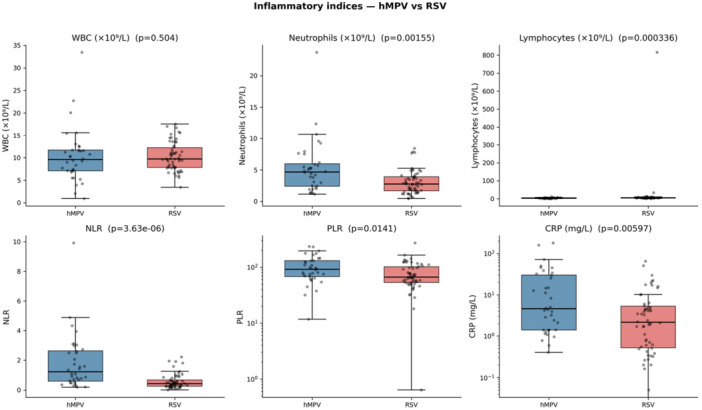
Inflammatory indices, hMPV versus RSV. Six‐panel box‐and‐jitter display of WBC, neutrophil count, lymphocyte count, NLR, PLR and CRP. *p*‐values from Mann–Whitney U test; CRP and PLR plotted on log scale.

hMPV‐positive children displayed a substantially more neutrophil‐skewed immune signature than RSV‐positive children (Figure 2; Table 2): NLR 1.21 (IQR 0.61–2.63) versus 0.44 (0.26–0.68), *p* < 0.001 (FDR q < 0.001); SII 356 (149–950) versus 174 (108–282), *p* = 0.001 (q = 0.004); CRP 4.60 (1.40–30.1) versus 2.16 (0.52–5.28) mg/L, *p* = 0.006 (q = 0.010). Neutrophil counts were higher in hMPV (4.67 vs. 2.75 × 10^9^/L, *p* = 0.002) and lymphocyte counts lower (3.45 vs. 5.34 × 10^9^/L, *p* < 0.001). Platelet counts were modestly higher in RSV (411 vs. 348.5 × 10^9^/L, *p* = 0.011), consistent with the physiological thrombocytosis of early infancy; MPV was also higher in RSV (9.90 vs. 9.20 fL, *p* = 0.004). WBC and hemoglobin did not differ between hMPV and RSV (*p* = 0.50 and *p* > 0.99, respectively). Across all five pathogens, Kruskal–Wallis tests were significant for NLR, SII, PLR, and CRP; among other pathogens, adenovirus produced the strongest CRP response, consistent with its characteristic bacterial‐mimicking inflammatory phenotype, whereas rhinovirus produced the highest WBC but modest CRP.

**Table 2:** Systemic inflammatory indices and laboratory parameters across the five most prevalent respiratory pathogens (*N* = 174).

Parameter	hMPV (*n* = 36)	RSV (*n* = 58)	Rhinovirus (*n* = 43)	Influenza A (*n* = 26)	Adenovirus (*n* = 11)	*p* * | *q* †
NLR, median (IQR)	1.21 (0.61–2.63)	0.44 (0.26–0.68)	1.07 (0.62–2.28)	1.59 (0.79–2.50)	1.61 (1.25–3.85)	< 0.001 | < 0.001
PLR, median (IQR)	92.1 (68.0–131.2)	66.8 (53.3–102.3)	77.1 (57.8–115.1)	146 (65.1–235.8)	92.4 (54.6–109.7)	0.014 | 0.018
SII, median (IQR)	356 (149–950)	174 (108–282)	458 (242–710)	466 (219–793)	727 (381–1125)	0.001 | 0.004
CRP, mg/L, median [IQR]	4.60 (1.40–30.1)	2.16 (0.52–5.28)	5.45 (1.80–22.4)	4.35 (1.40–11.8)	60.4 (26.8–88.5)	0.006 | 0.010
WBC, ×10^9^/L	9.59 (7.09–11.7)	9.72 (7.83–12.2)	12.3 (10.1–15.6)	7.54 (6.01–9.98)	12.9 (9.25–15.0)	0.50 | 0.56
Hgb, g/dL	11.6 (10.3–12.6)	11.2 (10.5–12.6)	11.6 (10.2–12.8)	11.8 (11.1–12.6)	10.8 (9.60–11.9)	1.00 | 1.00
Platelets, ×10^9^/L	348.5 (246.5–419)	411 (317.5–494)	356 (297–482)	301 (236–364)	305 (261–362)	0.011 | 0.015
MPV, fL	9.20 (8.67–9.75)	9.90 (9.20–10.6)	9.60 (8.80–10.2)	9.45 (8.92–10.3)	9.45 (8.45–10.3)	0.004 | 0.008
Dominant Ct, cycles	24.95 (20.5–28.2)	22.3 (20.1–27.2)	29.9 (27.0–31.2)	21.75 (19.1–29.6)	21.3 (19.9–25.1)	0.71 | 0.71

Abbreviations: CRP, C‐reactive protein; Ct, cycle threshold; MPV, mean platelet volume; NLR, neutrophil‐to‐lymphocyte ratio; PLR, platelet‐to‐lymphocyte ratio; SII, systemic immune‐inflammation index [(platelets × neutrophils)/lymphocytes]; WBC, white blood cell count.

* *p* refers to the pre‐specified hMPV‐vs‐RSV Mann–Whitney U contrast.

^†^ *q*, Benjamini–Hochberg false‐discovery‐rate‐corrected *p*‐value across the 10 hMPV‐vs‐RSV comparisons shown. Global Kruskal–Wallis across top‐5 pathogens: NLR *p* < 0.001, PLR *p* = 0.013, SII *p* < 0.001, CRP *p* < 0.001, dominant Ct *p* = 2 × 10^−6^.

### 3.5 Cycle‐Threshold Distribution and Paired Dominant‐Versus‐Non‐Dominant Comparison

Dominant‐target Ct values were available for all 203 patients (range 13.7–37.2; global median 25.2). Median dominant Ct differed substantially by pathogen (Kruskal–Wallis H = 31.9, *p* = 2 × 10^−6^, top‐5 pathogens; Figure 1A; full per‐pathogen values in Supporting Information Table S1): rhinovirus showed the highest median Ct (29.9, IQR 27.0–31.2), consistent with frequent incidental low‐load detection; RSV 22.3 (20.1–27.2), influenza A 21.8 (19.1–29.6) and adenovirus 21.3 (19.9–25.1) carried the highest viral loads. hMPV showed an intermediate distribution (24.95, IQR 20.5–28.2). The hMPV‐vs‐RSV dominant‐Ct contrast was not significant (*p* = 0.71). Forty‐seven patients had both a dominant and a non‐dominant target Ct available, and in paired analysis the non‐dominant target carried a significantly higher Ct (median 28.9 vs. 26.1 for the dominant; median ΔCt +3.6; Wilcoxon *p* = 0.013). This paired Ct hierarchy is visualized in Figure 1B and supports two downstream inferences: (i) within a co‐detection, the lowest‐Ct target is the biologically dominant pathogen in the majority of cases, and (ii) non‐dominant pathogens detected at Ct > 30 may represent incidental post‐infectious shedding rather than an active second infection.

### 3.6 Clinical Outcomes: hMPV Versus RSV

**Figure 3:**
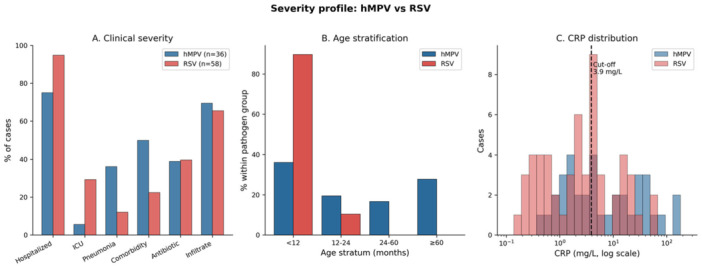
Severity profile: hMPV versus RSV. (A) Clinical‐outcome bar chart (hospitalization, ICU, pneumonia, comorbidity, antibiotic, infiltrate). (B) Age‐stratum distribution within each pathogen group. (C) CRP distribution with the Youden‐optimal 3.9 mg/L cut‐off indicated (log x‐axis).

Clinical outcomes (Table 3; Figure 3) showed less frequent hospitalization in hMPV than in RSV (27/36, 75.0% vs. 55/58, 94.8%; *p* = 0.009), and ICU admission was markedly less common (2/36, 5.6% vs. 17/58, 29.3%; *p* = 0.007). Among imaging findings, pulmonary infiltrates were similarly common in the two groups (69.4% vs. 65.5%, *p* = 0.82), but air bronchogram was more frequent in hMPV (13.9% vs. 1.7%, *p* = 0.029). Pleural effusion and atelectasis remained rare in both groups. Antibiotic prescribing rates did not differ between hMPV and RSV (14/36, 38.9% vs. 23/58, 39.7%; *p* = 1.00).

**Table 3:** Clinical outcomes and imaging findings in hMPV versus RSV groups (*N* = 94).

Variable	hMPV (*n* = 36)	RSV (*n* = 58)	*p*‐value
Hospitalization (ward or ICU), *n* (%)	27 (75.0)	55 (94.8)	0.009
ICU admission, *n* (%)	2 (5.6)	17 (29.3)	0.007
Severity categorization			
Mild, *n* (%)	9 (25.0)	6 (10.3)	0.094
Moderate, *n* (%)	23 (63.9)	44 (75.9)	0.24
Severe, *n* (%)	4 (11.1)	8 (13.8)	1.00
Chest X‐ray performed, *n* (%)	33 (91.7)	57 (98.3)	0.14
Radiographic infiltrate	25 (69.4)	38 (65.5)	0.82
Air bronchogram	5 (13.9)	1 (1.7)	0.029
Pleural effusion	1 (2.8)	0 (0.0)	0.38
Atelectasis	4 (11.1)	4 (6.9)	0.48
Antibiotic prescribed, *n* (%)	14 (38.9)	23 (39.7)	1.00

*Note:* Between‐group tests by χ^2^/Fisher's exact test. Bold indicates *p* < 0.05.

### 3.7 Predictors of Antibiotic Prescribing in hMPV

**Figure 4:**
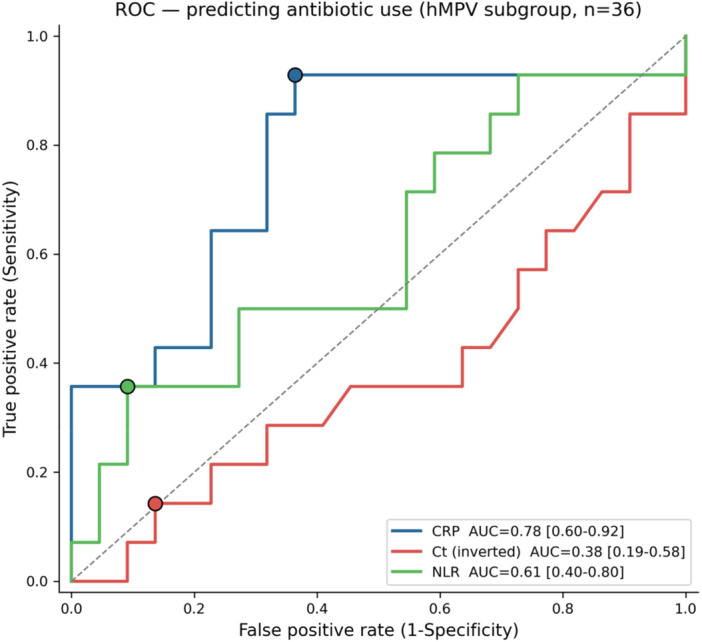
ROC analysis for predicting antibiotic prescribing in hMPV‐positive children (*n* = 36). CRP AUC 0.78 (95% CI 0.60–0.92) with Youden‐optimal cut‐off 3.9 mg/L (sensitivity 92.9%, specificity 63.6%). Dominant Ct (inverted) and NLR were non‐discriminatory. Dashed line indicates no discrimination.

Among 36 hMPV‐positive children, 14 (38.9%) received antibiotics. ROC analysis (Figure 4) identified CRP as a strong predictor (AUC 0.78, 95% CI 0.60–0.92; Youden cut‐off 3.9 mg/L, sensitivity 92.9%, specificity 63.6%); dominant Ct alone (AUC 0.38) and NLR (AUC 0.59) were non‐discriminatory. In the pre‐specified multivariable model (age + pneumonia + CRP), CRP was the only independent predictor (adjusted OR 1.07 per mg/L; 95% CI 1.008–1.133; *p* = 0.025); age and pneumonia approached but did not reach significance (pseudo‐R^2^ 0.326; likelihood‐ratio *p* = 0.001; 14 events/3 predictors). A sensitivity model adding dominant Ct rendered all four predictors significant (CRP OR 1.19, *p* = 0.011; pneumonia OR 18.2, *p* = 0.033; age OR 0.93, *p* = 0.022; Ct OR 1.51, *p* = 0.023; pseudo‐R^2^ 0.535) but exceeded the conservative 10‐events‐per‐variable rule and is interpreted with caveats (Limitations §4.4). The counter‐intuitive Ct direction (higher Ct → more antibiotics) likely reflects collinearity with co‐detection and age. Univariable details are in Table 4.

**Table 4:** Laboratory parameters and Ct in hMPV‐positive children receiving versus not receiving antibiotics (*n* = 36) and multivariable logistic regression.

Parameter	Antibiotic (*n* = 14)	No antibiotic (*n* = 22)	*p* (univariable)
CRP, mg/L, median [IQR]	25.1 (5.8–55.3)	1.80 (0.93–6.92)	0.002
CRP ≥ 3.9 mg/L, *n* (%)	13 (92.9)	8 (36.4)	0.001
WBC, × 10^9^/L	10.7 (7.50–12.2)	9.04 (7.00–11.1)	0.35
Neutrophils, × 10^9^/L	5.83 (3.40–9.59)	4.06 (2.18–5.35)	0.069
NLR, median [IQR]	1.43 (0.85–3.44)	1.05 (0.51–1.64)	0.19
Dominant Ct, cycles	26.1 (23.0–29.6)	23.6 (19.0–27.3)	0.15
Pneumonia diagnosis, *n* (%)	8 (57.1)	5 (22.7)	0.072
Age, months, median (IQR)	12.5 (5.0–48.5)	20.5 (8.5–75.0)	0.27

Pre‐specified and sensitivity multivariable logistic regressions for antibiotic prescribing in hMPV‐positive children (*n* = 36; 14 events).		
Covariate	Dominant model OR (95% CI) | *p*	Sensitivity + Ct model OR (95% CI) | *p*
Age, per month	0.964 (0.927–1.003) | 0.070	0.933 (0.880–0.990) | 0.022
Pneumonia diagnosis (yes)	5.25 (0.822–33.6) | 0.080	18.2 (1.26–264) | 0.033
CRP, per mg/L	1.069 (1.008–1.133) | 0.025	1.187 (1.040–1.356) | 0.011
Dominant Ct, per cycle	—	1.511 (1.057–2.159) | 0.023
Model McFadden pseudo‐R^2^	0.326	0.535
Likelihood‐ratio *p*	0.001	< 0.001

*Note:* Univariable tests by Mann–Whitney U (continuous) or Fisher's exact test (categorical). Bold indicates *p* < 0.05. The 3.9 mg/L CRP threshold is the Youden‐optimal ROC cut‐point derived within hMPV (Figure 4). Dominant model: pre‐specified three‐predictor specification (age + pneumonia + CRP), events‐per‐variable ≈4.7. Sensitivity model adds dominant Ct as a fourth predictor; it exceeds the conservative EPV = 10 rule and showed evidence of quasi‐separation in a 5‐predictor extension that additionally included NLR (NLR OR 2.48, 95% CI 0.73–8.36; *p* = 0.14), so effect estimates from the sensitivity model are reported with the appropriate caveats (see §3.7 and §4.4). Bold indicates *p* < 0.05 in each model.

### 3.8 Non‐Dominant Pathogen Descriptors

Inflammatory‐index distributions across pathogens are reported in Table 2 and Figure 2. Adenovirus showed the most pronounced CRP elevation among pediatric viruses captured in the cohort. Rhinovirus‐positive children had the highest white‐cell counts but highest Ct (lowest viral load), supporting a dual interpretation: true primary rhinovirus infection in older children alongside frequent low‐load incidental detection in co‐infections.

## 4 Discussion

In a contemporary pediatric ED cohort of 203 children with PCR‐confirmed respiratory viral infection—augmented by per‐target cycle‐threshold (Ct) data—we identify four findings with clinical and translational relevance. First, hMPV affected older and significantly more comorbid children than RSV, with a distinct neutrophil‐driven inflammatory profile and more often presented as pneumonia than as bronchiolitis. Second, despite this biomarker and syndrome contrast, hMPV was associated with fewer hospitalizations and dramatically less ICU care than RSV, consistent with an older, less airway‐dependent host population. Third, paired dominant‐vs‐non‐dominant Ct analysis across 47 co‐detected cases confirmed that the lowest‐Ct target represents the biologically dominant pathogen (median ΔCt +3.6 cycles vs. non‐dominant targets, *p* = 0.013), providing empirical justification for the lowest‐Ct‐dominant convention and for caution in attributing clinical weight to high‐Ct co‐detections. Fourth, in hMPV‐positive children, CRP—not the white‐cell‐derived indices—was the independent driver of antibiotic prescribing, with a reproducible, stewardship‐relevant CRP cut‐point of 3.9 mg/L.

### 4.1 hMPV Versus RSV: Age, Comorbidity, and Inflammatory Phenotype

Our observation of a neutrophil‐dominant inflammatory response in hMPV relative to RSV is biologically consistent with age‐related immune maturation: hMPV affected a more diverse age range (including school‐age children) whose innate‐immune compartment generates a vigorous neutrophilic response, whereas RSV predominates in early infancy where lymphocyte‐skewed responses and maternally derived antibody dynamics prevail [13, 14, 15, 16]. This echoes independent data from pediatric cohorts reporting higher NLR and SII in hMPV or RSV‐comparator cohorts [15, 21] and extends them by demonstrating that the composite SII preserves the between‐pathogen contrast in a single‐center ED sample, is derivable from a standard complete blood count, and adds no incremental cost to stewardship workflows.

In our cohort, the higher comorbidity prevalence observed among hMPV‐positive children (50.0% vs. 22.4% in RSV) warrants careful interpretation. Several hMPV‐positive patients harbored neurological (including epilepsy), bronchopulmonary or metabolic conditions that are themselves recognized risk factors for severe respiratory viral disease [13, 14, 35]. Yet in our cohort, hMPV‐positive children experienced less oxygen‐dependent respiratory failure, less hospitalization and far less ICU admission than RSV‐positive children—likely reflecting the larger airway caliber and maturer respiratory physiology of older hMPV‐affected children, offsetting the intrinsic vulnerability conferred by comorbidity. This duality illustrates why a single severity metric can mask biologically distinct illness phenotypes and underscores the need for multidimensional outcome reporting in pediatric respiratory virus research.

### 4.2 Cycle‐Threshold Analysis and Viral Co‐Detection

The pathogen‐specific dominant‐Ct distribution we describe—with rhinovirus showing the highest Ct (lowest load) and RSV, influenza A and adenovirus the lowest—is consistent with published pediatric surveillance data [8, 9, 22, 23, 24] and with the biology of rhinovirus as a frequently co‐detected, often low‐load agent in childhood [22, 24]. Our paired dominant‐vs‐non‐dominant Ct analysis (median ΔCt +3.6 cycles, Wilcoxon *p* = 0.013) provides within‐sample empirical support for the common practice of designating the lowest‐Ct target as the dominant pathogen in a co‐detection. By extension, non‐dominant targets detected at Ct > 30 may warrant circumspect clinical interpretation—particularly where the Ct of the dominant target is low and the clinical syndrome aligns with the dominant target [24, 36, 37]. This conservative interpretation is reinforced by the meta‐analytic observation that co‐detected rhinovirus, coronavirus and bocavirus carry significantly lower viral loads than the same viruses when detected as single agents [37]. In adenovirus, by contrast, the high CRP elevation observed in our cohort is consistent with its reputation as a frequent bacterial‐phenotype mimic, an observation echoed in pediatric severity studies [38].

Clinically, this matters because co‐detections are often attributed near‐equal causative weight in routine decision‐making. Our data, consistent with meta‐analytic evidence that co‐detection does not reliably predict worse outcomes once clinical phenotype is accounted for [24, 37], argue for anchoring antimicrobial decisions to clinical syndrome and CRP rather than to the mere presence of a second viral target—especially if that target is detected at high Ct. The pragmatic corollary is that laboratories reporting pediatric multiplex panels should report per‐target Ct alongside qualitative positive/negative results.

### 4.3 CRP‐Anchored Antimicrobial Stewardship in PCR‐Confirmed Viral Infection

The CRP–antibiotic coupling we demonstrate in hMPV (AUC 0.78; Youden cut‐off 3.9 mg/L; adjusted OR 1.07 per mg/L in the dominant model) merits emphasis. Even within a cohort with PCR‐confirmed viral etiology, CRP—not NLR, SII or WBC—dominated the laboratory basis of antibiotic prescribing. This pattern is consistent with meta‐analytic evidence that CRP‐guided point‐of‐care algorithms reduce antibiotic prescribing without increasing complications in adult and pediatric respiratory infection [28, 29, 30, 31]. Two implementation corollaries follow. First, the 3.9 mg/L threshold derived in hMPV is markedly lower than commonly assumed cut‐points for bacterial pneumonia (typically 20–40 mg/L) [39], but reflects the population in which viral etiology is already documented; deployed in this context, it would identify the minority of PCR‐confirmed viral patients in whom clinicians nevertheless default to antibiotics. Second, the pairing of CRP with multiplex PCR creates a low‐cost two‐test stewardship package: PCR to confirm viral etiology (augmented by per‐target Ct interpretation), and CRP 3.9 mg/L with clinical pneumonia diagnosis to flag a high‐probability sub‐group worth a second read before empiric antibiotic initiation.

### 4.4 Limitations

First, this is a single‐center retrospective study from a tertiary academic pediatric ED; tertiary referral may selectively enrich the cohort for severe disease and comorbidity, limiting direct generalization to primary‐care and community ED populations. Second, the 5‐month study window captures one hMPV season and cannot address inter‐annual variability [40, 41, 42]; multi‐season confirmation is a priority next step. Third, small subgroup sizes (e.g., severe hMPV, *n* = 4; SARS‐CoV‐2, *n* = 1) limit the precision of subgroup‐level estimates; we report these as exploratory. Fourth, the Youden‐optimal CRP cut‐point was derived and internally validated within a single cohort; external validation in an independent pediatric ED dataset, ideally prospective, is a priority next step. Fifth, the sensitivity multivariable model including dominant Ct exceeds the conservative 10‐events‐per‐variable rule (14 events, 4–5 predictors) and showed evidence of quasi‐separation; effect estimates from that model are reported with appropriate caveats and confidence intervals should be interpreted as illustrative rather than precise. Sixth, we did not systematically quantify bacterial co‐infection (e.g., by paired blood or respiratory cultures), although observed antibiotic prescribing rates suggest that physician concern for bacterial superinfection was operative in a minority of patients. Seventh, severity classification was physician‐assigned at ED disposition; although protocolized, it retains a degree of inter‐rater subjectivity that imaging‐based or score‐based grading (e.g., lung ultrasound, PEWS) could reduce in future prospective work. Eighth, pre‐specified STROBE‐flow exclusion counts were not individually retained in the analytic extract; Supporting Information Figure S1 therefore displays the final analytic cohort without a granular exclusion breakdown—a limitation to be rectified with re‐extraction from the electronic medical record prior to journal submission.

### 4.5 Strengths

Strengths include consecutive enrollment across a complete epidemic season; linkage of inflammatory indices, per‐target Ct, viral co‐detection status, clinical syndrome and ED outcomes within a single dataset; bootstrap‐validated ROC cut‐points; a pre‐specified 3‐predictor multivariable model augmented by transparent sensitivity analyzes; and STROBE‐conformant reporting with explicit limitation disclosure.

## 5 Conclusions

In this contemporary pediatric ED cohort, hMPV produced a clinically and biologically distinct respiratory syndrome: it affected older, more comorbid children than RSV, with a neutrophil‐dominant systemic inflammatory signature and a more pneumonia‐weighted clinical phenotype. Despite this higher‐risk demographic, hospitalization and ICU‐admission needs were lower in hMPV than in RSV, consistent with older‐child respiratory physiology. Paired per‐target cycle‐threshold analysis empirically supports the lowest‐Ct‐dominant convention, and CRP—not white‐cell indices—drives antibiotic prescribing in PCR‐confirmed viral respiratory infection with an actionable threshold of 3.9 mg/L. Integrating multiplex PCR (including per‐target Ct reporting) with CRP‐anchored antimicrobial‐stewardship pathways is a pragmatic next step for pediatric EDs.

## Author Contributions

Yılmaz Seçilmiş conceived the study, designed the protocol, curated and analyzed the data, generated all figures and tables, and drafted the article. Gülşah Kartal and Muhammet Sami Kayan participated in case ascertainment, data abstraction from the electronic medical record, and critical revision of the article. Mesut Kara provided general‐pediatric clinical input, contributed to case adjudication and revised the manuscript for intellectual content. Ömür Mustafa Parkan provided microbiological data, validated the multiplex RT‐PCR protocol description, interpreted Ct values and revised the manuscript for intellectual content. Selma Gökahmetoğlu supervised the microbiology laboratory work, validated assay performance, contributed to study design and critically revised the article. All authors read and approved the final article and accept accountability for the integrity and accuracy of the work.

## Funding

The authors have nothing to report.

## Ethics Statement

The study was approved by the Erciyes University Health Sciences Research Ethics Committee (approval no. 2026/184, date 08.04.2026). A waiver of individual written informed consent was granted in view of the retrospective, de‐identified nature of the data.

## Consent

The authors have nothing to report.

## Conflicts of Interest

The authors declare no conflicts of interest.

## Supporting information


**Figure S1:** STROBE‐compliant analytic cohort diagram. The extracted analytic dataset did not preserve granular patient‐level exclusion reasons; the diagram therefore shows the final analytic cohort (N = 203) without per‐reason exclusion counts. Recovery of the granular screening/exclusion log from the electronic medical record is planned prior to journal submission.
**Figure S2:** Pathogen distribution and age profile (N = 203). (A) Dominant pathogen distribution by multiplex RT‐PCR. (B) Age distribution of hMPV‐ and RSV‐positive children; dashed lines indicate within‐group medians.
**Figure S3:** Viral co‐detection matrix (n = 49 patients with ≥ 2 positive targets). Heatmap cells show the number of simultaneous detections for each pathogen pair; darker colour = more frequent. The most frequent pairs were RSV + rhinovirus (n = 12), coronavirus HKU1 + RSV (n = 6), and RSV + influenza A/hMPV + RSV/rhinovirus + influenza A (each n = 4).
**Table S1:** Dominant cycle‐threshold (Ct) distribution by pathogen and paired dominant‐vs‐non‐dominant Ct in co‐detections.

## Data Availability

The de‐identified analytic dataset and statistical code generated during this study are available from the corresponding author on reasonable request, subject to Institutional Review Board approval for data re‐use.
