# Supplementary material for: Human Metapneumovirus Versus Respiratory Syncytial Virus in the Pediatric Emergency Department: Inflammatory Indices, Cycle‐Threshold Profiles and Outcomes
Source: J Med Virol. 2026 Aug 10;98(8):e71099. doi: 10.1002/jmv.71099 (PMC13455020; doi:10.1002/jmv.71099)
Supplement: Supplementary file 1 [file JMV-98-e71099-s001.docx]

**Supplementary Table S1.** Dominant cycle-threshold (Ct) distribution by pathogen and paired dominant-vs-non-dominant Ct in co-detections.

| **Pathogen** | **n (dominant)** | **Ct median [IQR]** | **Ct range** | **Ct mean ± SD** |
| --- | --- | --- | --- | --- |
| RSV | 58 | 22.3 [20.1–27.2] | 15.5–37.2 | 24.1 ± 5.0 |
| Rhinovirus | 43 | 29.9 [27.0–31.2] | 21.0–35.8 | 29.1 ± 3.5 |
| hMPV | 36 | 24.95 [20.5–28.2] | 14.7–33.8 | 24.4 ± 5.6 |
| Influenza A | 26 | 21.75 [19.1–29.6] | 13.7–34.2 | 23.5 ± 5.9 |
| Adenovirus | 11 | 21.3 [19.9–25.1] | 18.1–35.3 | 23.1 ± 4.9 |
| Influenza B | 10 | 25.6 [22.0–28.1] | 14.8–34.3 | 25.5 ± 5.8 |
| Parainfluenza | 7 | 24.7 [20.9–28.0] | 16.8–30.5 | 24.3 ± 4.9 |
| Seasonal CoV (all) | 10 | 25.0 [22.1–30.4] | 18.5–34.5 | 25.1 ± 5.1 |
| Bocavirus | 1 | 16.8 (single) | 16.8 | — |
| SARS-CoV-2 | 1 | 28.0 (single) | 28.0 | — |
| All dominant targets | 203 | 25.2 [21.1–28.6] | 13.7–37.2 | 25.1 ± 5.3 |

*Kruskal–Wallis across top-5 pathogens p=2×10⁻⁶ (Figure 3A). Paired dominant-vs-non-dominant Ct analysis (n=47 pairs with both targets' Ct available; Figure 3B): dominant median Ct 26.1, non-dominant median 28.9, median ΔCt +3.6 cycles, Wilcoxon signed-rank p=0.013. The ΔCt hierarchy empirically supports the lowest-Ct-dominant convention used in this study (see Methods §2.4 and Results §3.5). Lower Ct indicates higher viral load; per manufacturer validation, samples were considered positive at Ct <40.*

**Figures**

**Supplementary Figure S2.** Pathogen distribution and age profile (N=203). (A) Dominant pathogen distribution by multiplex RT-PCR. (B) Age distribution of hMPV- and RSV-positive children; dashed lines indicate within-group medians.

**Supplementary Figure S3.** Viral co-detection matrix (n=49 patients with ≥2 positive targets). Heatmap cells show the number of simultaneous detections for each pathogen pair; darker colour = more frequent. The most frequent pairs were RSV + rhinovirus (n=12), coronavirus HKU1 + RSV (n=6), and RSV + influenza A / hMPV + RSV / rhinovirus + influenza A (each n=4).

**Supplementary Material**

**Supplementary Figure S1.** STROBE-compliant analytic cohort diagram. The extracted analytic dataset did not preserve granular patient-level exclusion reasons; the diagram therefore shows the final analytic cohort (N=203) without per-reason exclusion counts. Recovery of the granular screening/exclusion log from the electronic medical record is planned prior to journal submission.

**Supplementary Checklist S1. STROBE statement (items 1–22)**

A completed STROBE checklist mapping each of the 22 items to the corresponding page and section of the main manuscript is submitted as a separate supplementary file. In brief: Title/Abstract (items 1a–1b) → Title page; Introduction (items 2–3) → §1; Methods (items 4–12) → §2; Results (items 13–17) → §3; Discussion (items 18–21) → §4; Other information (item 22) → Declarations.
